# Association between the serum creatine kinase level and all-cause mortality in centenarians: a prospective cohort study in China

**DOI:** 10.3389/fendo.2026.1773804

**Published:** 2026-05-11

**Authors:** Xinyan Gong, Xiaowei Cheng, Qingtao Zhang, Yue Niu, Xiang Yu, Yuwei Ji, Baolong Wang, Hongyan Hu, Qiao Zhu, Miao Liu, Yali Zhao, Yao He, Zehao Zhang, Zeyu Qu, Xiangmei Chen, Yizhi Chen, Zhe Feng

**Affiliations:** 1 Department of Nephrology, Hainan Hospital of Chinese People’s Liberation Army (PLA) General Hospital, Academician Chen Xiangmei of Hainan Province Kidney Diseases Research Team Innovation Center, Sanya, China; 2 Senior Department of Nephrology, Chinese People’s Liberation Army (PLA) General Hospital, State Key Laboratory of Kidney Diseases, National Clinical Research Center for Kidney Diseases, Beijing, China; 3 Department of Laboratory Medicine, Hainan Hospital of Chinese People’s Liberation Army (PLA) General Hospital, Sanya, China; 4 Central Laboratory, Hainan Hospital of Chinese People’s Liberation Army (PLA) General Hospital, Sanya, China; 5 Department of Anti-Nuclear, Biological, Chemical Medicine, Graduate School, Chinese People’s Liberation Army General Hospital, Beijing, China; 6 Institute of Geriatrics, Beijing Key Laboratory of Aging and Geriatrics, National Clinical Research Center for Geriatric Diseases, Second Medical Center, State Key Laboratory of Kidney Diseases, Chinese People’s Liberation Army (PLA) General Hospital, Beijing, China; 7 The Second School of Clinical Medicine, Southern Medical University, Guangzhou, China; 8 Hainan Medical University, Haikou, China; 9 Sanya Nephrology Medical Quality Control Center, Sanya, China

**Keywords:** all-cause mortality, centenarians, creatine kinase, longevity, muscle mass

## Abstract

**Background:**

Muscle mass (MM) has a strong correlation with all-cause mortality. However, its assessment is not easily accessible and expensive. Serum creatine kinase (CK) has been proposed as a solution, but its association with mortality remains unclear. This study aimed to investigate the association between the serum CK level and all-causes mortality among centenarians in China.

**Methods:**

This prospective cohort study included Chinese centenarians residing in the community between June 2014 and December 2016. All-cause mortality was analyzed according to serum CK level using restricted cubic spline (RCS) analysis, Cox regression analysis, Kaplan–Meier curves, the log-rank test, and subgroup analysis.

**Results:**

In total, 949 centenarians were eligible for participation and were followed up for a median of 29.4 months (IQR 14.5, 51.7). During the study period, 92.9% of the study participants died. RCS analysis revealed an inverse J-shaped relationship between the serum CK level and the risk of death. Mortality was 34.3% higher when the serum CK level was in the range of 8–66 U/L (Q1–Q2) than in the range of 66–192 U/L (Q3–Q4) (HR = 1.343 in multivariable analysis; 95%CI: 1.173–1.538; P<0.001). Kaplan–Meier analysis showed a significantly shorter median survival in the low-CK group (Q1–Q2) compared to the high-CK group (Q3–Q4). (26 months vs 36 months; P<0.001, log-rank test).

**Conclusions:**

Among community-dwelling centenarians, serum CK level was independently and inversely associated with all-cause mortality in an inverse J−shaped manner and may serve as a practical biomarker for physiological status and survival.

## 1 Introduction

Adequate nutritional screening and assessment are essential for maintenance of nutritional status and survival in the elderly. Low muscle mass (MM) is highly prevalent in the elderly population and correlates with poor outcomes (1–5). Therefore, MM is considered to be an important indicator when assessing the likelihood of survival in elderly individuals (6). Sarcopenia is a progressive and generalized skeletal muscle disorder that is associated with increased likelihood of adverse outcomes including falls, fractures, physical disability and mortality. In 2019, the Asian Working Group for Sarcopenia (AWGS 2019) published an updated consensus specifically for Asian populations, which is more suitable for elderly individuals in Asia. According to the AWGS 2019 criteria, sarcopenia is diagnosed by the presence of both low muscle quantity and either low muscle strength or low physical performance. Severe sarcopenia is defined as the coexistence of low muscle mass, low muscle strength, and low physical performance (7). Furthermore, in patients with chronic kidney disease, sarcopenia is a frequent complication (8) and a risk factor for adverse clinical outcomes, including mortality (9). However, assessment of MM using bioimpedance techniques or a muscle scanner is complex, not readily accessible, and expensive, which means that our ability to measure MM as an indicator of sarcopenia is limited in routine clinical practice (10–12). In contrast, measurement of the serum creatine kinase (CK) level has been reported to be a cost-effective, noninvasive, and simple technique for estimating MM (13–15).

CK is an important enzyme in the body and is directly related to muscle contraction (16). Its main function is the reversible catalytic generation of phosphocreatine and adenosine diphosphate from creatine and adenosine triphosphate (17). Although CK has been the focus of considerable attention in the assessment of MM in the elderly population, whether it has a direct relationship with mortality in this age group has been unclear. Mortality in the elderly has been attributed more often to factors such as age (18), chronic disease (19–21), and lifestyle (22–24) than to the CK level. Furthermore, although previous studies have tended to focus on the clinical significance of serum CK levels above the normal range, research on the relationship between mortality and CK levels within the normal range in the elderly has been limited. Therefore, the aim of this study was to evaluate the relationship between serum CK levels in the normal range and all-cause mortality in centenarians.

## 2 Methods

### 2.1 Study design and patients

This study analyzed data from the prospective observational China Hainan Centenarian Cohort Study, which collected baseline data for 1002 centenarians who were residing in the community between June 2014 and December 2016 (25). The date of death was confirmed in each case by the Hainan Provincial Civil Affairs Bureau, which is responsible for the monthly pensions paid to individuals aged 80 years and older and checks that centenarians are alive at least monthly. The study was approved by the Ethics Committee of the Chinese People’s Liberation Army (PLA) General Hospital (No. 301HNLL-2016-01) and performed in accordance with the Declaration of Helsinki and its subsequent amendments. All study participants provided written informed consent before enrollment.

### 2.2 Sample size determination

This prospective cohort study was designed to investigate the association between serum CK levels and all-cause mortality in centenarians. Based on previous studies concerning biomarkers and mortality in the oldest-old population, a clinically meaningful hazard ratio (HR) of 1.20 to 1.50 was assumed. With a type I error (α) of 0.05 (two-sided) and a statistical power of 80%, the minimum required sample size was calculated to be approximately 300 participants. Considering the advanced age, high expected mortality rate, and potential loss to follow-up among centenarians, we finally enrolled 949 eligible participants, which was sufficiently large to detect the targeted association.

### 2.3 Data collection

A multidisciplinary clinical team at Hainan Hospital of Chinese PLA General Hospital performed comprehensive health assessments for the centenarian cohort using structured interviews. Standardized questionnaires were used to systematically collect demographic and anthropometric data (including age, sex, ethnic background, weight, height, educational attainment, marital status, smoking status, alcohol consumption, hypertension, diabetes mellitus (DM), coronary heart disease (CHD), and a range of serological measures, including standard blood counts and biochemical tests).

​Hypertension was diagnosed if systolic blood pressure was ≥140 mmHg and/or diastolic blood pressure was ≥90 mmHg. DM was defined according to the 1999 World Health Organization diagnostic criteria for diabetes (26). Body mass index (BMI) was calculated as weight in kilograms divided by height in meters squared. Blood samples were collected from the study participants by an experienced nurse and sent to the laboratory at the Hainan Hospital of the Chinese PLA General Hospital for analysis. The CK level was quantified by dynamic measurement of the absorbance resulting from formation of nicotinamide adenine dinucleotide phosphate at 340/660 nm, which is proportional to the activity of CK in a human serum sample. This method is a modification of the International Federation of Clinical Chemistry method used to measure the catalytic concentrations of enzymes on a Beckman Coulter analyzer. The normal range is between 2 U/L and 200 U/L.

### 2.4 Statistical analysis

Data that were normally distributed are shown as the mean ± standard deviation and were compared between groups using the independent samples *t*-test or analysis of variance. Data that were unevenly distributed are shown as the median (interquartile range [IQR]) and were compared between groups using the Mann–Whitney *U* test or Kruskal–Wallis *H* test. Categorical variables are shown as the number and percentage and were compared between groups using the chi-squared test.

Restricted cubic spline (RCS) analysis is widely used to study nonlinear associations. In this study, RCS analyses were performed to assess the associations between all-cause mortality and serum CK levels as continuous variables in both unadjusted and multivariate-adjusted models. A proportional hazards assumption test was performed by RCS analysis before performing the Cox regressions. The CK levels were grouped by IQR as follows: Q1, 8≤CK<48 U/L; Q2, 48≤CK<66 U/L; Q3, 66≤CK<92 U/L; and Q4, 92≤CK ≤ 192 U/L. Both unadjusted and adjusted Cox regression analyses were performed. The initial regression assessed the hazard ratio (HR) and 95% confidence interval (CI) for mortality, and the subsequent regression was adjusted for age, sex, ethnicity, marital status, BMI, educational background, smoking status, alcohol consumption, DM, hypertension, and CHD. The distribution of time to death was analyzed by Kaplan–Meier curves and the log-rank test. Subgroup analyses were performed to determine whether there were any variables that could potentially affect the relationship between the CK level and all-cause mortality. The *P*-values for interaction were evaluated using likelihood ratio tests. All statistical analyses were performed using R software (version 4.3.3; https://www.r-project.org/). A *P*-value of <0.05 was considered statistically significant.

## 3 Results

### 3.1 Characteristics of the study population at baseline and during follow-up

Fifty-three centenarians were excluded because their baseline information was incomplete or their CK level was above the normal range, leaving data for 949 centenarians available for analysis. **Table 1** and **Supplementary Table 1** show the characteristics of the study population according to the CK quartile at baseline. The average age at baseline was 102 years (IQR 101, 104), and 81.9% of the centenarians were female. Approximately 73.8% had hypertension; however, the prevalence rates of DM and CHD were low (9.3% and 4.2%, respectively). Mortality was 92.9% (882/949) during a median follow-up of 29.4 months (IQR 14.5, 51.7). The median CK level was 66 U/L (IQR 48, 92).

**Table 1:** Characteristics of the study population according to creatine kinase quartile at baseline.

Variable	Overall	Q1 [8, 48)	Q2 [48, 66)	Q3 [66, 92)	Q4 [92, 192]	P value
N	949	224	249	236	240	
Age, years	102.00(101.00–104.00)	102.00(101.00–104.00)	102.00(101.00–104.00)	102.00(101.00–104.00)	102.00(101.00–104.00)	0.147
Female, %	777 (81.87)	191 (85.27)	207 (83.13)	195 (82.63)	184 (76.67)	0.090
Follow-up time, months	29.40(14.50,51.70)	23.20(11.35,36.83)	28.00(12.80,50.30)	35.80(15.07,55.73)	37.40(19.05,61.73)	< 0.001
Death, %	882 (92.94)	218 (97.32)	228 (91.57)	221 (93.64)	215 (89.58)	0.009
Ethnicity						0.624
Han, %	843 (88.83)	204 (91.07)	221 (88.76)	206 (87.29)	212 (88.33)	
Other, %	106 (11.17)	20 (8.93)	28 (11.24)	30 (12.71)	28 (11.67)	
Marital status						0.400
Married, %	99 (10.43)	30 (13.39)	22 (8.84)	23 (9.75)	24 (10.00)	
Separation/Divorce/ Widowhood, %	850 (89.57)	194 (86.61)	227 (91.16)	213 (90.25)	216 (90.00)	
Education						0.481
No, %	865 (91.15)	202 (90.18)	235 (94.38)	213 (90.25)	215 (89.58)	
Elementary school, %	64 (6.74)	18 (8.04)	11 (4.42)	17 (7.20)	18 (7.50)	
Junior high school and above, %	20 (2.11)	4 (1.79)	3 (1.20)	6 (2.55)	7 (2.92)	
Smoking						0.998
Never, %	845 (89.04)	202 (90.18)	221 (88.76)	210 (88.99)	212 (88.33)	
Past, %	70 (7.38)	15 (6.70)	19 (7.63)	17 (7.20)	19 (7.92)	
Now, %	34 (3.58)	7 (2.12)	9 (3.61)	9 (3.81)	9 (3.75)	
Alcohol						0.293
Never, %	782 (82.40)	182 (81.25)	206 (82.73)	202 (85.59)	192 (80.00)	
Past, %	73 (7.69)	24 (10.71)	17 (6.83)	12 (5.08)	20 (8.33)	
Now, %	94 (9.91)	18 (8.04)	26 (10.44)	22 (9.32)	28 (11.67)	
Hypertension, %	700(73.76)	159 (71.98)	179 (71.89)	183 (77.54)	179 (74.58)	0.366
DM, %	88 (9.27)	18 (8.04)	20 (8.03)	19 (8.05)	31 (12.92)	0.167
CHD, %	40 (4.21)	8 (3.57)	9 (3.61)	12 (5.08)	11 (4.58)	0.807
BMI, kg/m^2^	18.00(16.04, 19.98)	17.86(16.04, 19.10)	17.65(15.72, 20.05)	18.42(16.43, 20.23)	18.40(16.54, 20.23)	0.008
CK, U/L	66.00(48.00,92.00)	38.00(32.00,43.00)	57.00(52.00,61.00)	78.00(71.00,84.00)	116.00(103.00,133.25)	< 0.001

CK, creatine kinase; BMI, body mass index; DM, diabetes mellitus; CHD, coronary heart disease; Q1–Q4, quartiles 1 to 4. Units for CK quartiles are U/L. Quartile intervals: “[“ denotes a closed interval (value included), “)” denotes an open interval (value not included).

### 3.2 RCS analyses of serum CK levels

**Figure 1:**
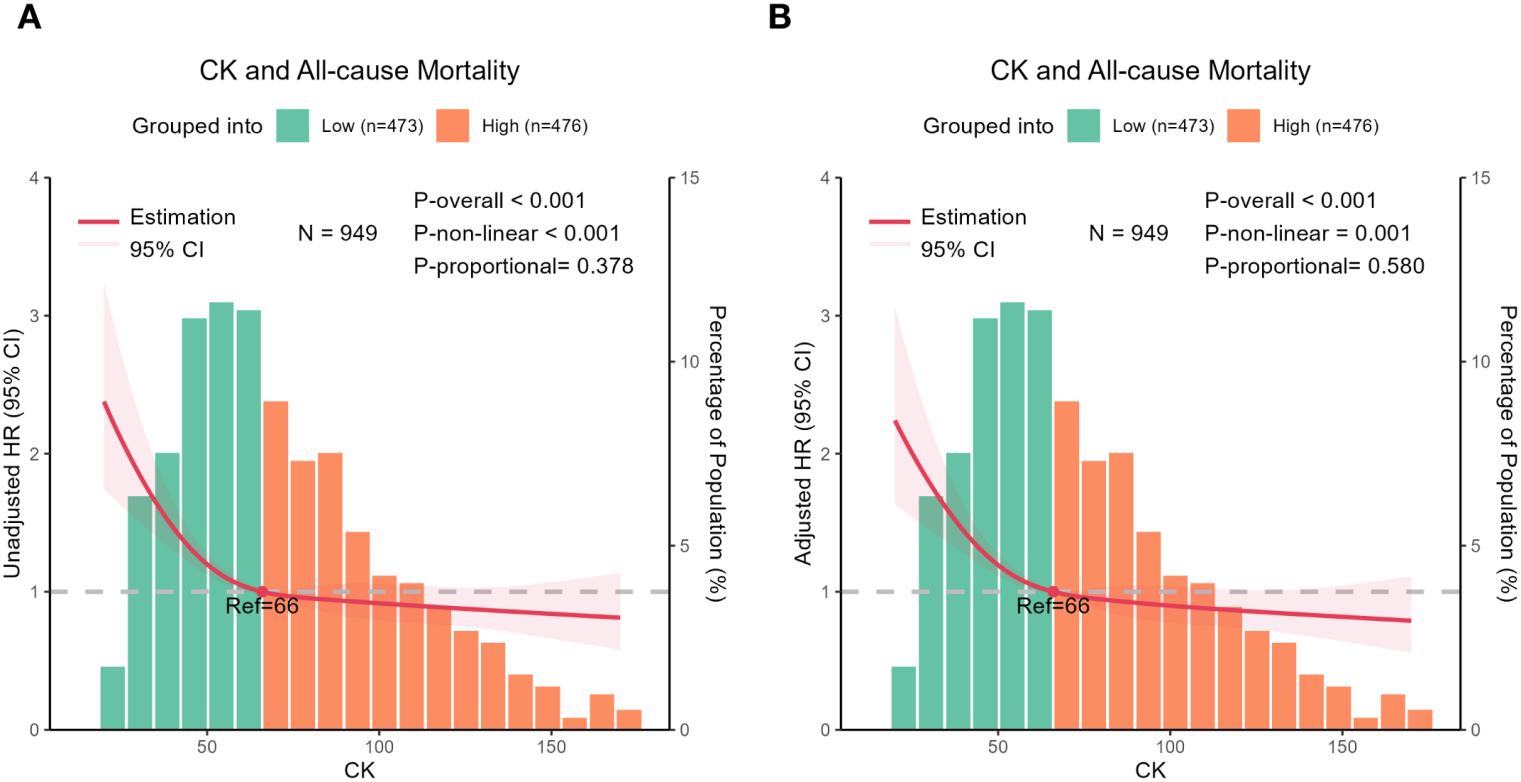
Restricted cubic spline (RCS) analysis of all-cause mortality according to creatine kinase (CK) level. The solid red line indicates the hazard ratio and the shaded areas represent the 95% confidence interval. The horizontal coordinates indicate the CK level, the left vertical coordinates indicate the hazard ratio for all-cause mortality, and the right vertical coordinates indicate the percentages of centenarian participants with those CK levels for the histogram. Q1, Q2, Q3, Q4 represent quartiles. The multivariate analysis was adjusted for age, sex, ethnicity, marital status, body mass index, educational level, smoking status, alcohol consumption, diabetes mellitus, hypertension, and coronary heart disease. The reference cut-off for serum CK level was a median value of 66 U/L. The RCS curves in both unadjusted **(A)** and multivariable-adjusted **(B)** figures show a smooth reverse J-shaped pattern, indicating that centenarians with a CK level below 66 U/L have a significantly increased risk of mortality. In centenarians, the risk of mortality decreased as the serum CK level increased. However, the risk of all-cause mortality stabilized when the median serum CK level was higher than 66 U/L. **Figures 1**-**3** were created by the authors.

Serum CK levels were significantly associated with all-cause mortality in both the unadjusted model (**Figure 1A**) and the multivariable-adjusted model (**Figure 1B**) (*P*_overall_ *<*0.05 for both). Both the unadjusted and adjusted models demonstrated an inverse J-shaped relationship between the serum CK level and the risk of death (*P*_nonlinear_ *<*0.001 and =0.001, respectively). These findings indicated that all-cause mortality in centenarians decreased as the serum CK level increased and this inverse relationship stabilized beyond a median CK level of 66 U/L.

### 3.3 Univariate and multivariate Cox analyses and Kaplan–Meier curves based on the serum CK level

The proportional hazards assumptions were confirmed for all the Cox regression models used in this study. In both univariate and multivariate analyses, the lower the serum CK level, the higher the mortality risk (**Table 2**). For every 10-U/L increase in the serum CK concentration, the risk of death was reduced by 5.7% in univariate analysis (95% CI 3.7–7.7) and by 5.8% in multivariate analysis (95% CI 3.7–7.8). The group with the highest IQR (Q4) was used as the reference. The risk of mortality was higher in the Q1 group than in the Q4 group, with the highest risk demonstrated for Q1 (HR 1.779, 95% CI 1.471–2.152, *P* < 0.001 in univariate analysis; HR 1.811, 95% CI 1.491–2.199, *P* < 0.001 in multivariate analysis). When the study population was divided into two groups based on the median serum CK level (Q1–Q2 vs. Q3–Q4), there was a significant correlation between the CK level and mortality in both univariate analysis (HR 1.351, 95% CI 1.183–1.542, *P* < 0.001) and multivariate analysis (HR 1.343, 95% CI 1.173–1.538, *P* < 0.001). This finding indicated that overall mortality increased by 34.3% in centenarians with a CK level below 66 U/L (Q1--Q2).

**Table 2:** Univariate and multivariate Cox proportional hazards analyses of the association of the serum creatine kinase level with all-cause mortality in centenarians.

Terms	Count	Univariate analysis		Multivariate -adjusted analysis	
CK	949	HR	P	HR	P
Continuous, per 10 U/L		0.943 (0.923–0.963)	< 0.001	0.942 (0.922–0.963)	< 0.001
Grouped into quartiles					
Q1 [8, 48)	224	1.779 (1.471–2.152)	< 0.001	1.811 (1.491–2.199)	< 0.001
Q2 [48, 66)	249	1.242 (1.031–1.497)	0.023	1.274 (1.054–1.541)	0.012
Q3 [66, 92)	236	1.163 (0.964–1.403)	0.116	1.230 (1.016–1.488)	0.034
Q4 [92, 192]	240	1 (Reference)		1 (Reference)	
P for trend			< 0.001		< 0.001
Grouped by medium value					
Q1-Q2 [8,66)	473	1.351 (1.183, 1.542)	< 0.001	1.343 (1.173, 1.538)	< 0.001
Q3-Q4 [66,192]	476	1 (Reference)		1 (Reference)	
P for trend			< 0.001		< 0.001

CK, creatine kinase; HR, hazard ratio; Q1–Q4, quartiles 1 to 4. Units for CK quartiles are U/L. Quartile intervals: “[“ denotes a closed interval (value included), “)” denotes an open interval (value not included).

Tables were generated from original data of the present study.

**Figure 2:**
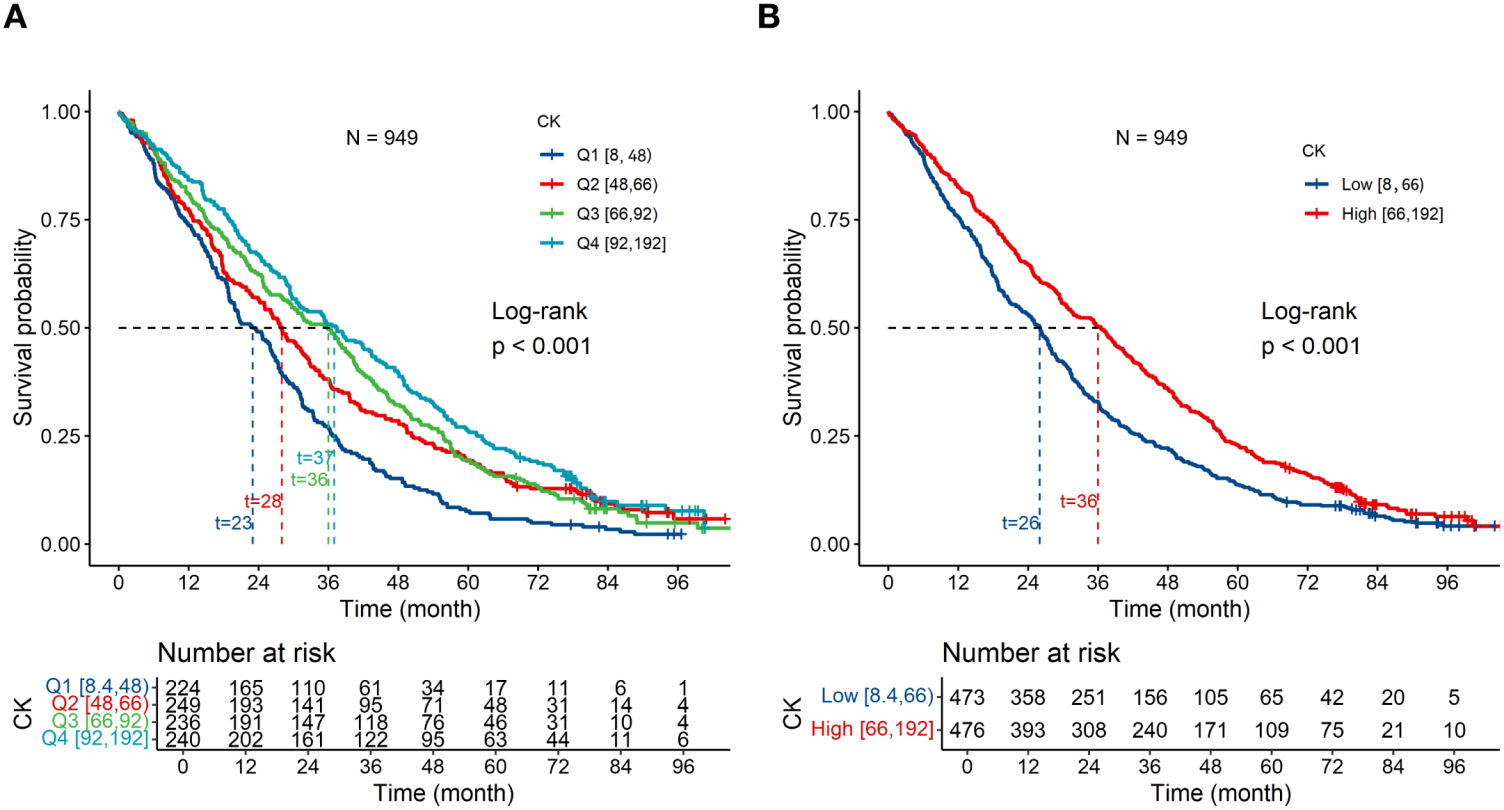
Kaplan–Meier survival curves and log-rank tests for the association of all-cause mortality with the serum creatine kinase (CK) level. Kaplan–Meier survival curves revealed significant associations of lower CK levels with shorter survival. The median survival time was significantly shorter for participants in the Q1 group than for those in the Q4 group (23 months versus 37 months, *P* < 0.001, log-rank test) **(A)**. The median survival duration was also shorter for centenarians in the lower CK group (Q1–Q2) than for those in the higher CK group (Q3–Q4) (26 months versus 36 months, *P* < 0.001, log-rank test) **(B)**.

The Kaplan–Meier curves revealed a significant correlation between a higher serum CK level and a longer survival time in centenarians. Median survival was significantly shorter in the Q1 group than in the Q4 group (23 months versus 37 months, *P* < 0.001, log-rank test) (**Figure 2A**) and was also shorter in the Q1–Q2 group than in the Q3–Q4 group (26 months versus 36 months, *P* < 0.001, log-rank test) (**Figure 2B**).

### 3.4 Subgroup analyses of variables that could influence the association between CK and all-cause mortality

**Figure 3:**
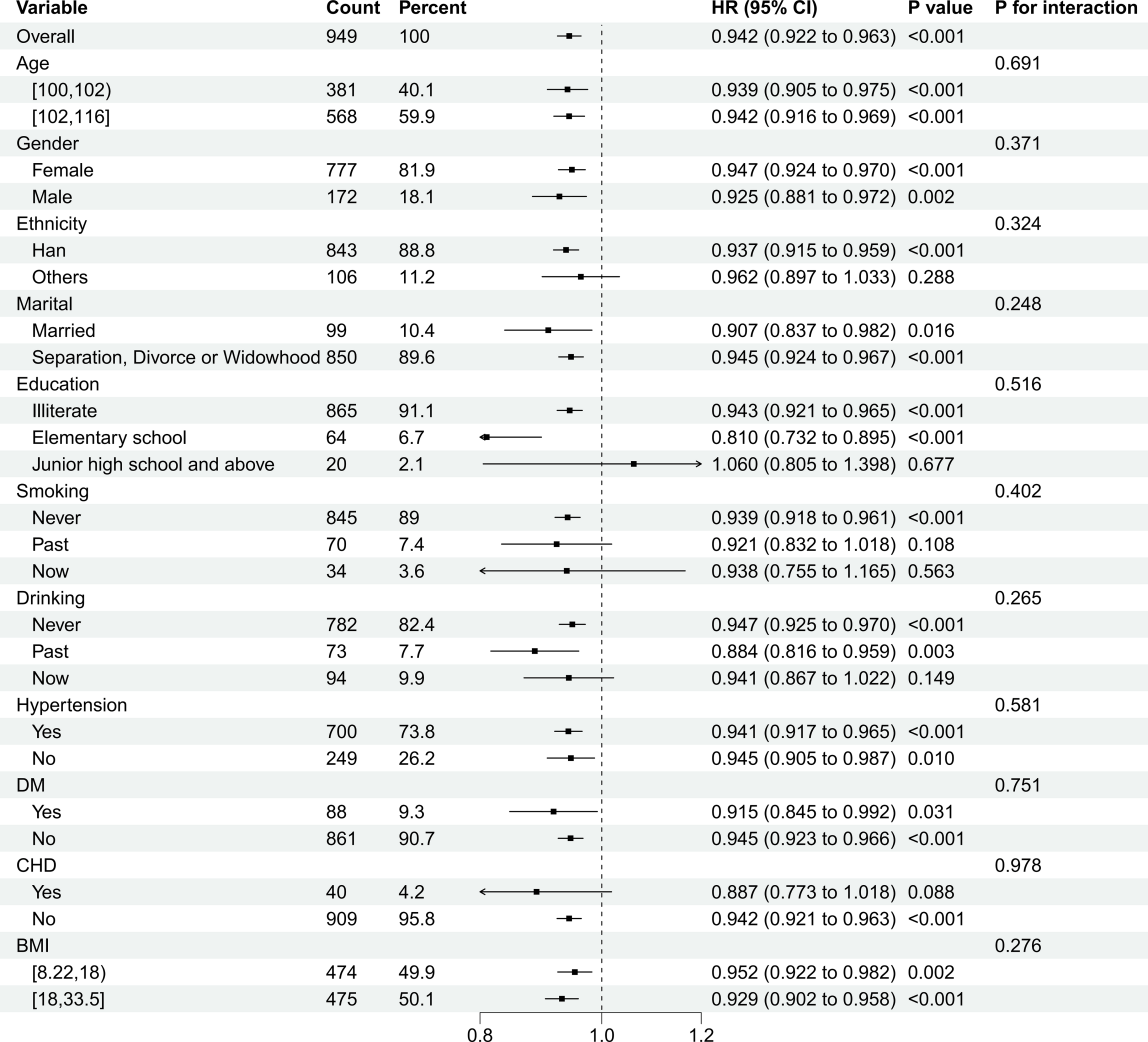
Subgroup analysis of the association between the serum creatine kinase (CK) and all-cause mortality. The forest plot presents the hazard ratios with 95% confidence intervals across subgroups stratified by age, sex, ethnicity, marital status, body mass index, education, smoking status, alcohol consumption, diabetes mellitus, hypertension, and coronary heart disease. P-values for interactions between subgroups are provided. Each 10-U/L increase in CK was associated with a significantly decreased risk of mortality in centenarians, with an adjusted hazard ratio of 0.94 (95% confidence interval 0.92–0.96, *P* < 0.001). The multivariate analysis was adjusted for age, sex, ethnicity, marital status, body mass index, education, smoking status, alcohol consumption, diabetes, hypertension, and coronary heart disease. The association between a reduced serum CK level and increased risk of mortality remained consistent across all subgroups, with no significant interaction effects detected (all *P*_interaction_ values were >0.05).

No significant interaction effects were observed across subgroups (P interaction >0.05 for all), indicating that the association between CK and mortality was consistent across the strata examined (**Figure 3**).

## 4 Discussion

CK is mainly synthesized and released into the blood by skeletal muscle and participates in intracellular energy metabolism by converting phosphocreatine to ATP to provide rapid energy for muscle contraction. CK content differs substantially among fiber types, and type II muscle fibers are rich in cytosolic CK and represent the primary source of serum CK (27, 28). Based on this tissue origin, the serum CK level is positively correlated with a number of parameters that directly reflect MM (29, 30). A cross-sectional study of 1,086 inpatients with type 2 DM revealed that CK was inversely correlated with low MM and positively correlated with the skeletal muscle index value (13). Serum CK levels were also negatively correlated with sarcopenia in a cross-sectional study of patients with osteoarthritis in Japan (14). Another cross-sectional study from Germany found that loss of skeletal MM was associated with a decreased serum CK level in older adults (31). Overall, the findings of these studies suggest a strong correlation between CK and MM, whereby decreasing CK levels are associated with loss of MM.

With advancing age, type II muscle fibers undergo reductions in number and size (32), which directly leads to decreased overall synthesis and release of CK, resulting in a gradual age-related decline in serum CK levels. Many studies have demonstrated an inverse relationship between advancing age and the serum CK level. A systematic review by Aleksovska et al. confirmed an age-dependent decline in CK reference values (33). The reference range has been defined as 59–314 U/L in young Chinese men aged 20–29 years and 54–293 U/L in their middle-aged counterparts aged 30–79 years. There is a similar decline in the reference range for Chinese women from 37–178 U/L in those aged 20–49 years to 34–155 U/L in those aged 60–79 years (34). This pattern is consistent globally; for example, the mean serum CK level has been found to be higher in young US men of mean age 28.9 ± 8.1 years than in middle-aged Norwegian men of mean age 43 ± 4 years and even higher than in older Norwegian men aged 64 ± 8 years (237.8 U/L vs 189 U/L and 147 U/L, respectively) (35, 36). The same age-related downward trend has been observed in women, with a median serum CK value of 248 U/L (95% CI 184–340) in Israeli young women of mean age 20.2 ± 2.9 years, decreasing to 95 U/L (95% CI 36–349) in middle-aged women of mean age 45 ± 7 years in the Netherlands and to a median of only 40 U/L (95% CI 18–184) in older Swiss women of mean age 85 ± 13 years (36–38). In our study, the median CK level was 66 U/L (IQR 48, 92) in centenarians, which also follows this age-related downward pattern.

Sarcopenia is defined as an age-related decline in skeletal MM and function (39). In elderly individuals, sarcopenia is considered to be a reliable marker of frailty and a poor prognosis and is often the result of a combination of physiological changes associated with aging and disease (4, 40). In a prospective cohort study in Italy, data were collected from 197 individuals aged 80–85 years living in the Sirente area (41). The primary outcome was all-cause mortality over a 7-year follow-up period. The risk of death from any cause was greater in subjects with sarcopenia than in those without sarcopenia (HR 2.32, 95% CI 1.01–5.43). In that study, sarcopenia was associated with mortality independent of age and other clinical or functional variables. In a study performed at a trauma center between 2009 and 2010, low MM and/or sarcopenia were associated with increased mortality in patients aged ≥65 years (42). Muscle tissue in the arms and legs, known as appendicular lean mass, makes up 75% of the skeletal muscle in the body and is required for walking and other body functions. A cohort study that included 487 individuals aged 65 years concluded that higher appendicular lean mass was associated with a lower risk of death (43). In a 10-year prospective cohort study that included 418 older adults in Brazil, low MM, assessed by calf, arm, arm muscle, and corrected arm muscle circumference, was associated with an increase in all-cause mortality (44). All of these studies suggested that there is a strong association between MM and mortality in elderly individuals, with a gradual increase in mortality in response to decreasing MM.

Previous studies have investigated the relationship between CK levels and mortality risk, but most focused on abnormally elevated CK or specific patient population. One study reported that a low serum CK level was associated with an increased risk of death in a population with chronic kidney disease (13). On the contrary, a prospective cohort study from Massachusetts in the USA found that a CK level >1000 U/L at admission independently predicted a higher risk of in-hospital mortality in critically ill patients with coronavirus disease 2019 (45). A retrospective study of patients with acute coronary syndrome presenting to an emergency department found that an elevated serum CK level independently predicted a higher risk of in-hospital death (46). Similarly, in a cross-sectional study from Japan, an elevated CK level was independently associated with increased 72-hour mortality in patients with a random plasma glucose level ≥500 mg/dL (47). A retrospective Japanese study also demonstrated that an elevated CK level was significantly associated with both an increased risk of acute kidney injury and higher mortality or a poor prognosis in patients who were hospitalized with seasonal influenza (48). However, the relationship between normal-range serum CK levels and all-cause mortality in centenarians has rarely been studied. Our study demonstrated an inverse J-shaped association between the serum CK level and the risk of mortality in this age group. As serum CK level declined, there is a progressive increase in the risk of death.

The divergent results between our study and previous reports can be explained by major differences in study populations and CK ranges. The association between serum CK and all-cause mortality lies in its ability to objectively reflect the basic physiological status of the body. Physiologically, the age-related reduction in serum CK within the normal range essentially indicates decreased skeletal muscle mass and impaired muscle function. Insufficient muscle mass further leads to reduced metabolic efficiency, diminished physical reserve, and weakened immune function, increasing the risk of adverse events such as infection and organ dysfunction, ultimately raising the risk of all-cause mortality. The present study included community-dwelling centenarians without acute severe diseases, all of whom had CK levels within the normal clinical range. Therefore, the observed association between lower CK and higher mortality risk does not reflect acute muscle injury or severe pathological conditions indicated by abnormally elevated CK. Instead, lower CK levels within the normal range indicate insufficient muscle reserve and relatively weak physiological function, which reduce the body’s ability to resist disease and stress, thereby indirectly increasing mortality risk.

Based on the actual results obtained in this study (HR = 1.343, 95% CI: 1.173–1.538), we performed a power calculation. The results confirmed that 949 participants provided adequate statistical power to detect the association between CK levels and mortality.

This study has several limitations. First, direct measurement of MM was not performed in the present study; instead, serum CK was used as a surrogate marker reflecting muscle mass and function. Therefore, although we demonstrated a relationship between the CK level and mortality, the lack of direct MM assessment may be considered a limitation. Future studies should incorporate more accurate methods for assessment of body composition, such as bioelectrical impedance analysis or dual-energy X-ray absorptiometry to better quantify MM and confirm our present findings. Second, our study participants were drawn mainly from the Chinese Han population and were the oldest adult age group. Therefore, our results might not be applicable to other ethnic populations or age groups and need to be confirmed in multi-ethnic and age-specific cohorts in the future. Third, although we observed a significant association between the CK level and mortality, potential confounders (e.g., medication, thyroid dysfunction, or acute illness) should be considered when interpreting our findings. Fourth, we were unable to monitor dynamic changes in the CK level and explore the relationship between dynamic CK and all-cause mortality in centenarians. Finally, we did not investigate the correlation between cause-of-death-specific mortality and the serum CK level because of unavailability of records concerning causes of death, such as cancer and cardiovascular disease.

## 5 Conclusions

Among community-dwelling centenarians, serum CK level may indirectly reflect diminished muscle reserve and poorer general physiological status and thus was independently and inversely associated with all-cause mortality in an inverse J-shaped manner.

## Data Availability

The original contributions presented in the study are included in the article/**Supplementary Material**. Further inquiries can be directed to the corresponding authors.
