# Supplementary material for: Association between the serum creatine kinase level and all-cause mortality in centenarians: a prospective cohort study in China
Source: Front Endocrinol (Lausanne). 2026 May 11;17:1773804. doi: 10.3389/fendo.2026.1773804 (PMC13199071; doi:10.3389/fendo.2026.1773804)
Supplement: Supplementary file 1 [file Table1.docx]

**Supplementary Table** **1** Characteristics of the study population based on CK median at baseline

| **Variables** | **Overall** | **Q1-Q2[8,66)** | **Q3-Q4[66, 192]** | **P value** |
| --- | --- | --- | --- | --- |
| N | 949 | 473 | 476 |  |
| Age,years | 102.00(101.00,104.00) | 102.00 (101.00–104.00) | 102.00 (101.00–104.00) | 0.604 |
| Female,% | 777 (81.88) | 398 (84.14) | 379 (79.62) | 0.085 |
| Follow-up time, months | 29.40(14.50, 51.70) | 26.00(12.40, 43.10) | 36.50(17.25, 57.30) | < 0.001 |
| Death,% | 882 (92.94) | 446 (94.29) | 436 (91.60) | 0.135 |
| Ethnicity |  |  |  | 0.372 |
| Han,% | 843 (88.83) | 425 (89.85) | 418 (87.82) |  |
| Other,% | 106 (11.17) | 48 (10.15) | 58 (12.18) |  |
| Marital status |  |  |  | 0.647 |
| Married,% | 99 (10.43) | 52 (10.99) | 47 (9.87) |  |
| Separation/Divorce/Widowhood,% | 850 (89.57) | 421 (89.01) | 429 (90.13) |  |
| Education |  |  |  | 0.294 |
| No education,% | 865 (91.15) | 437 (92.39) | 428 (89.92) |  |
| Elementary school,% | 64 (6.74) | 29 (6.13) | 35 (7.35) |  |
| Junior high school and above,% | 20 (2.11) | 7 (1.48) | 13 (2.73) |  |
| Smoking habits |  |  |  | 0.920 |
| Never,% | 845 (89.04) | 423 (89.43) | 422 (88.66) |  |
| Past,% | 70 (7.38) | 34 (7.19) | 36 (7.56) |  |
| Now,% | 34 (3.58) | 16 (3.38) | 18 (3.78) |  |
| Alcohol consumption |  |  |  | 0.466 |
| Never,% | 782 (82.40) | 388 (82.03) | 394 (82.78) |  |
| Past,% | 73 (7.70) | 341(8.67) | 32 (6.72) |  |
| Now,% | 94 (9.91) | 44 (9.30) | 50 (10,50) |  |
| Hypertension,% | 700 (73.76) | 338 (71.46) | 362 (76.05) | 0.125 |
| Diabetes,% | 88 (9.27) | 38 (8.03) | 50 (10.50) | 0.230 |
| Coronary heart disease,% | 40 (4.21) | 17 (3.59) | 23 (4.83) | 0.431 |
| Body mass index, kg/m^2^ | 18.00(16.04, 19.98) | 17.78(15.83, 19.64) | 18.42(16.46, 20.23) | 0.001 |
| Creatine kinase, U/L | 66.00(48.00,92.00) | 48.00(39.00, 57.00) | 92.00(78.00, 116.00) | <0.001 |
